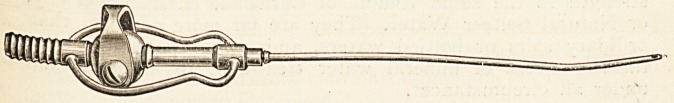# Notes on Preparations for the Sick

**Published:** 1900-03

**Authors:** 


### Notes on Preparations for the Sick.

---

**Vitogen.**—G. F. HARVEY Co., Saratoga Springs, N.Y. Agents for Great Britain: THOS. CHRISTY & Co., London.—This is a light pinkish yellow non-odorous powder of antiseptic character. It is one of the many substitutes for iodoform, and its use is said to be unattended by any poisonous results. Its chemical composition is not stated.

---

**The "Multi" Dressing (Registered) of Hartmann's Absorbent Cotton Wool.**—THE SANITARY WOOD WOOL Co., LD., London.—The dressing is put up in convenient form, but the specimen of wool sent us is not quite as absorbent as others we have used. The generally reliable character of Messrs. Hartmann's products is well known.

**Nasal Duct Irrigator.**—White and Wright, Liverpool.—We have received the adjoining woodcut, which represents a cannula for irrigating the lachrymal sac and nasal duct. It has been used in its present form for two years by its designer, Mr. Hugh E. Jones, of Liverpool, and has given perfect satisfaction. The chief points about the instrument are the following:—1. It is made of silver. 2. The cannula is closed at the distal end and two catheter eyes cut as near the end as possible. In this way the use of the stilette is avoided, while it is practically impossible to injure the delicate mucous membrane of the duct while passing the cannula. 3. The outside diameter does not exceed that of Bowman's original No. 6 probe, so that it is only necessary to divide half the length of the canaliculus in order to pass the instrument. 4. There is a tap at the proximal end of the instrument whereby the flow from the irrigator (which should be at least 6 feet above the patient's head) may be controlled by the hand which handles the instrument. If preferred the cannula can be obtained without the tap, and a clamp used on the irrigator tube.

---

Many new **Tabloids** have been issued by the firm of Messrs. Burroughs, Wellcome & Co.

**Tabloids.**—Bismuth Subgallate gr. v. is insoluble in water or alcohol; it is therefore best administered in a dry state as a compressed tabloid. In various kinds of dyspepsia and diarrhœa these tabloids should be a very convenient and useful form.

Aloes and Iron, sugar coated, gr. iv.
Colocynth Comp. compressed, gr. iv.
Zinc Iodide, gr. ij.
Cannabinol, gr. j.

Cascara Sagrada, gr. iij.
Cascara Sagrada, gr. j.

These tabloids of the dry extract are prepared with the idea that the stronger one may be taken once, twice, or even thrice daily for habitual constipation until the habit of a regular action of the bowels is established, when the dose may be reduced to the weaker tabloid taken once daily. They are issued both plain and sugar coated.

Potassium Chloride, grs. xx.

These are recommended as preferable to common table salt for the use of the gouty. They are easily crushed into powder at the table and taken with the food.

**"Tabloid" Artificial Effervescent Mineral Water Salts.**—These "Tabloid" Mineral Water Salts form pleasant effervescent draughts, one in a given quantity of water being equal in strength to the same volume of Carlsbad, Kissingen, Vichy, or Natural Seltzer Water. They are far more compact than ordinary salts or bottled waters, and render possible a continuous course of mineral water treatment in all lands and under all circumstances.

---

**Soloid.**—BURROUGHS, WELLCOME & CO.—Potassium Permanganate gr. iij. and Alum gr. v. These compound soloids are a very convenient form for the easy production of an efficient antiseptic and astringent solution for many purposes.

---

**Glycerine Enule.**—BURROUGHS, WELLCOME & CO.—A rectal suppository containing 95 per cent. of pure anhydrous glycerine in a patent protective sheath.

---

**Nepenthe Suppositories.**—FERRIS & CO., Bristol.—These are made in three strengths, equivalent to $\frac{1}{2}$ grain, $\frac{1}{4}$ grain, and $\frac{1}{6}$ grain of morphia.

---

THE ELBERFELD FARBEN FABRIKEN COMPANY have sent us the following samples of their excellent preparations:

**Somatose.**—A tasteless and odourless albumose food.

**Trional.**—A most reliable hypnotic, now given most conveniently dissolved in aërated water.

**Tannigen.**—An intestinal astringent.

**Europhen.**—An odourless substitute for iodoform.

**Aristol.**—A cicatrisant of the first order.

**Analgen.**—A specific for malaria and anti-rheumatic.

**Losophan** (Triiodometacresol).—Antiparasitic.

**Iodothyrine.**—One tablet is equivalent to five grains of fresh gland.

**Lycetol.**—A uric-acid solvent.

**Heroin.**—An excellent substitute for codeine.

**Creasotal.**—Creasote carbonate.

**Duotal.**—Guaiacol carbonate.

**Aspirin.**—A new substitute for salicylic acid, with no unpleasant by-effects.

---

**Egg Emulsion of Cod Liver Oil.**—PARKE, DAVIS & CO., London.—Contains 40 per cent. of Cod Liver Oil emulsified with egg and flavoured with Brandy. It has the aspect of a perfect emulsion, and has the quality of permanency. It readily mixes with water, milk, or wine.

**Elixir Saw Palmetto and Santal Compound.**—Parke, Davis & Co., London.—Is a very elegant preparation of several useful drugs which have an established reputation for the purpose of overcoming vesical, urethral, and prostatic irritability.

---

**D.C.L. Malt Extract.**—The Distillers' Company, Limited, Edinburgh.—Malt extracts contain besides the diastase of the malt and predigested starch (maltose and dextrine), some of the albuminates and salts of the barley. They are not only nourishing in themselves, but they help to digest other nourishment as well. This company claim to be the largest firm of distillers in the world, and to have facilities at their disposal for making the very best malt extract. It appears to be all that can be desired.

---

## Figures and Tables

**Figure f1:**